# Safety and efficacy of biolimus-eluting stent with biodegradable polymer: insights from EINSTEIN (Evaluation of Next-generation drug-eluting STEnt IN patients with coronary artery disease) Registry

**DOI:** 10.1590/S1679-45082013000300015

**Published:** 2013

**Authors:** Cristiano Freitas de Souza, Anwar Mohamed El Mouallem, Fábio Sândoli de Brito, Alexandre Antônio Cunha Abizaid, Breno Oliveira Almeida, Amanda Gonçalves Almeida, Teresa Cristina Dias Cunha Nascimento, Marco Antonio Perin, Adriano Caixeta

**Affiliations:** 1 Hospital Israelita Albert Einstein, Sao Paulo, SP, Brazil.

**Keywords:** Biocompatible events, Pharmacological stents, Drugdelivery systems, Polymers/chemical synthesis, Sirolimus/analogs & derivatives, Coronary artery disease/therapy

## Abstract

**Objective:**

To evaluate the incidence of major adverse cardiac events (cardiac death, or acute myocardial infarct, or target vessel revascularization) at one year in “real world” patients.

**Methods:**

The EINSTEIN registry is an observational, prospective, single center study that consecutively included 103 patients (152 lesions) treated with the Biomatrix™ stent, a biolimus A9-eluting stent with biodegradable polymer.

**Results:**

The mean age was 65.0±12.4 years; male gender represented 83.5% of the patients; and 37.9% of them were diabetic. At one-year, major adverse cardiac events occurred in 11.7% of the patients, including 2.9% of cardiac death, 4.9% of with non ST- segment elevation acute myocardial infarction, and 3.9% of target vessel revascularization. Stent thrombosis occurred in only 1% (1 patient) at one-yearfollow-up.

**Conclusion:**

The present Registry suggests that new generation biolimus A9 drug-eluting stents are safe and effective in a “real world”, all-comers patients, showing low rates of major cardiac adverse events on long-term follow-up.

## RESUMO

**objetivo:**

Avaliar a incidência de eventos adversos cardíacos maiores (morte cardíaca, infarto agudo do miocárdio ou necessidade de nova revascularização do vaso-alvo) em 1 ano em pacientes do “mundo real”.

**Métodos:**

O registro EINSTEIN é um estudo observacional prospectivo, unicêntrico, que incluiu consecutivamente 103 pacientes (152 lesões) tratados com o *stent* Biomatrix™, eluidor de biolimus A9 e polímero biodegradável.

**Resultados:**

A média das idades foi de 65,0±12,4 anos; o gênero masculino representou 83,5% dos pacientes; e 37,9% deles eram diabéticos. Em 1 ano, eventos adversos cardíacos maiores ocorreram em 11,7% dos pacientes, incluindo 2,9% de morte cardíaca, 4,9% de infarto agudo do miocárdio sem supradesnivelamento do segmento ST e 3,9% de revascularização do vaso-alvo. Trombose de *stent* esteve presente em apenas 1% (1 paciente) após o seguimento clínico de 1 ano.

**Conclusão:**

O presente registro sugere que os novos *stents* farmacológicos eluidores de biolimus A9 com polímero biodegradável são seguros e eficazes em pacientes da prática clínica diária, com baixas taxas de eventos adversos cardíacos maiores no longo prazo.

## INTRODUCTION

Since its incorporation into medical practice a decade ago, drug-eluting stents (DES) have been used in various clinical and anatomical scenarios due to their reduction in restenosis rates. Compared to bare-metal stents, first generation DES were more effective in decreasing neointimal hyperplasia, which consequently led to a reduction in late luminal loss and binary restenosis^(1–3)^. Subsequently, however, concerns have been raised about the increased late and very late thrombosis with first generation DES^(4–6)^. Although the mechanism of thrombotic events is not yet fully elucidated, hypersensitivity reaction to the permanent/non degradable polymer is one of the potential explanation in this complex equation^(7,8)^. Thus, a novel generation of DES such as Biomatrix™ (Biosensors International, Morges, Switzerland) has been emerged with the aim to diminish stent thrombosis as well as other safety complications related to first generation DES. One of the Biomatrix™ main features is the presence of a biodegradable polymer comprised of polylactic acid. This polymer is applied only on its abluminal surface which, after a total drug release in 6 to 9 months, is metabolized into carbon acid and water. Biomatrix™ is eluted with e biolimus A9 drug, an immunosuppression and antiproliferative compound of the same family as sirolimus, but with solubility ten times higher. In the randomized LEADERS study, the Biomatrix™ stent proved to be safer than the first-generation DES Cypher™^(9)^. However, the long-term clinical results with Biomatrix™ stent in a real world, all- comers patients are scarce.

## OBJECTIVE

The primary endpoint of the EINSTEIN (Evaluation of Next-generation drug-eluting STEnt IN patients with coronary artery disease) registry is to evaluate the incidence of major adverse cardiac events (MACE). MACE was defined as the composite of cardiac death, acute myocardial infarct, or target vessel revascularization at 30 days, 6 months, 12 months, and annually up to 3 years follow-up. The secondary objectives were (1) procedure success, defined as the technical success with residual stenosis ≤30%; (2) occurrence of in-hospital MACE; (3) individual events of death, acute myocardial infarct, target vessel revascularization, and stent thrombosis at 30 days, 6 months, and 12 months.

## METHODS

### Patients and study design

The EINSTEIN registry was a prospective and singlecenter registry that enrolled consecutive and nonselected patients underwent percutaneuos coronary intervention (PCI) with Biomatrix™ stent implantation. From October 2008 to December 2010, a total of 103 patients with 152 coronary lesions were treated with Biomatrix™ stent at the Department of Interventional Cardiology at Hospital Israelita Albert Einstein (HIAE), in São Paulo. Among them, 101 (98.0%) completed one-year follow-up, representing the subject of analysis of this manuscript. Clinical follow-up was prespecified at 30 days, 6 months, 1 year, and yearly thereafter for a total 3 years in all patients. In order to demonstrate the daily clinical practice usefulness of the Biomatrix™ stent, the inclusion criteria were permissive, enrolling all-comers patients >18 years underwent elective or emergency PCI. Since it is an observational and retrospective analysis of our database, all signed informed Consent Forms were waived by the institutional Ethics Committee. The protocol was approved by the Ethics Committee of HIAE under the Nº. 11/1530 (CAAE: 0038.0.028.000-11).

### Coronary intervention procedure and anti-aggregation protocol

The antiplatelet protocol consisted dual antiplatelet therapy with aspirin and clopidogrel or prasugrel prior stenting. After the procedure, aspirin 100mg/day and indefinitely, clopidogrel 75mg/day or prasugrel 10mg/day were recommended for at least 12 months. The number of stents implanted and techniques used were at physician's discretion.

### Definitions

Cardiac death was defined as any death not attributable to a non-cardiac cause. Myocardial infarction was defined as when at least one of the following criteria was present: elevation of CPK-MB over three times the upper limit of normal; electrocardiogram with a new pathological Q-wave (≥0.04 seconds) in at least two contiguous leadings with positive CPK-MB. Target vessel revascularization was defined as any attempt or successful PCI or coronary artery bypass grafting (CABG) percutaneous of the target-vessel. Stent thrombosis was classified according to the Academic Research Consortium (ARC) definition as definite, probable or possible. As per protocol, definite stent thrombosis was defined as a thrombus detected by angiography or total occlusion within the stented vessel at the time of clinically driven angiography for ischemia; or by pathological confirmation of an acute thrombosis in a patient with acute coronary syndrome. Probable stent thrombosis was defined as any death not attributed to a noncardiac cause or any myocardial infarction in the target vessel territory in the absence of documental angiographic stent patency. Possible stent thrombosis was defined as unexplained death after 30 days^(10)^.

Clinical follow-up information was collected by telephone contact or medical visit at 30 days, 6 months, 12 months, and then annually thereafter.

### Collection and management of data

Preparation and implementation of the registry were under the responsibility of the Department of Cardiovascular Intervention at HIAE.

### Statistical analysis

In the descriptive analysis, categorical variables are presented as counts and percentages. Continuous variables are expressed as mean±standard deviation. One-year outcome are summarized as Kaplan-Meier curves and compared by log-rank statistical test. A p value of <0.05 was considered statistical significant. All statistical analysis were performed by the Statistical Package for the Social Science (SPSS) 13.0 software.

## RESULTS

### Clinical and procedure characteristics

The baseline clinical characteristics are shown in table 1. Mean age was 65.0±12.4 years, with a predominance of male gender (83.5%). Among risk factors for coronary artery disease (CAD), we highlight the high prevalence of diabetes mellitus (37.9%), systemic arterial hypertension (75.7%), and hypercholesterolemia (70.9%). The most prevalent clinical presentation was stable angina/silent ischemia (47.6%), followed by unstable angina (23.3%). Primary PCI for acute myocardial infarction was performed in 11.7% of the patients.

**Table 1:** Baseline clinical characteristics, risk factors, clinical presentation and medications

		n=103
Age, years		65.07±12.49
Male gender, % (n/total)		83.5 (86/103)
Body mass index, kg/m^2^		27.54±4.17
Hypertension, % (n/total)		75.7 (78/103)
Hypercholesterolemia, % (n/total)		70.9 (73/103)
Smoking, % (n/total)		25.2 (26/103)
*Diabetes mellitus*, % (n/total)		37.9 (39/103)
	Insulin-dependent	5.8% (6/103)
Prior AMI, % (n/total)		12.6 (13/103)
Prior TCA, % (n/total)		38.8 (40/103)
Prior MR, % (n/total)		18.4 (19/103)
Clinical presentation, % (n/total)		
	Stable angina/silent ischemia	47.6 (49/103)
	Acute coronary syndrome	52.4 (54/103)
	Unstable angina	23.3 (24/103)
	AMI with no ST segment elevation	17.5 (18/103)
	AMI with ST segment elevation	11.7 (12/103)
Family history positive for CAD, % (n/total)		33.0 (34/103)
Coronary disease, % (n/total)		
	Single arterial	36.9 (38/103)
	Double arterial	33.0 (34/103)
	Triple arterial	30.1 (31/103)
Number of lesions per patient		1.62±0.74
Use of aspirin, % (n/total)		
	Inhospital	56.3 (58/103)
	30 days	90.7 (78/86)
	6 months	90.4 (75/83)
	1 year	80.2 (77/96)
Use of clopidogrel, % (n/total)		
	Inhospital	43.7 (45/103)
	30 days	95.3 (82/86)
	6 months	92.8 (77/83)
	1 year	79.2 (76/96)

AMI: acute myocardial infarct; TCA: transluminal coronary angioplasty; MR: myocardial revascularization; CAD: coronary artery disease.

Procedural and angiographic characteristics are shown in tables 2 e 3. Most patients treated were multivessell (63%), including one third with three-vessel disease. Treatment included *de novo* lesions, intra-stent restenosis (12.7%), and treatment of bifurcation lesions (16.4%).

**Table 2:** Initial angiographic characteristics

		n=152
Treated artery, % (n/total)		
	Anterior descendent	49.7 (75/151)
	Right coronary	18.5 (28/151)
	Circumflex	23.8 (36/151)
	Saphenous bypass	6.6 (10/151)
	Mammary bypass	0.7 (1/151)
	Non-protected coronary trunk	0.7 (1/151)
Location of the lesion, % (n/total)		
	Ostial	13.8 (21/152)
	Proximal	49.3 (75/152)
	Medial	23.7 (36/152)
	Distal	13.2 (20/152)
Type of lesion, % (n/total)		
	*De novo* stenosis	87.3 (131/150)
	Intra-stent restenosis	12.7 (19/150)
Initial target vessel reference values		
	Reference diameter of vessel, mm	2.43±0.60 (n=148)
	Minimum luminal diameter, mm	0.94±0.54 (n=145)
	Diameter of the stenosis, %	62.41±20.41 (n=148)
	Extension of the lesion, mm	17.40±11.91 (n=137)
Characteristics of the target vessel, % (n/total)		
	Angulation>45°	6.6 (10/152)
	Angulation 45-90°	4.6 (7/152)
	Angulation>90°	2.0 (3/152)
	Prior thrombus	5.9 (9/152)
	Moderate/severe tortuosity	6.6 (10/152)
	Moderate/severe calcification	23.7 (36/152)
	Ulceration	2.0 (3/152)
	Intimal flap	2.0 (3/152)
	Ectasis	1.3 (2/152)
	Bifurcation	16.4 (25/152)
Prior TIMI, % (n/total)		
	0/1	7.9 (12/152)
	2	6.6 (10/152)
	3	84.2 (128/152)

TIMI: thrombolysis in myocardial infarction.

**Table 3:** Post procedure angiographic characteristics

		n=152
Factors related to the procedure		
	Diameter of the stent, mm	3.09±0.43 (n=148)
	Total length of the stent, mm	23.19±11.97 (n=148)
	Stent length by lesion length ratio, mm/mm	1.57±0.76 (n=119)
	Number of stents per lesion	1.16±0.50 (n=152)
Final reference values of the target vessel		
	Reference diameter of the vessel, mm	2.91±0.61 (n=148)
	Minimum luminal diameter, mm	2.23±0.58 (n=148)
	Diameter of the stenosis, %	23.41±9.77 (n=148)
	Minimum luminal intra-stent diameter, mm	2.48±0.53 (n=148)
	Diameter of the intra-stent stenosis, %	15.57±7.21 (n=148)
Final TI MI, % (n/total)		
	0/1	1.3 (2/152)
	2	0.7 (1/152)
	3	98.0 (149/152)
Final findings of the target lesion, % (n/total)		
	Thrombus	0.0 (0/152)
	Distal embolization	0.7 (1/152)
	Acute occlusion	0.0 (0/152)
	No reflow	0.0 (0/152)
	Perforation	0.7 (1/152)
	Dissection	0.7 (1/152)

TIMI: thrombolysis in myocardial infarction.

### Clinical follow-up

Table 4 displays the major clinical endpoints during hospitalization, at 30 days, and12 months. Clinical follow-up was completed in 98% of the patients.

**Table 4:** In hospital, 30 days, and 12 months follow-up and the respective cumulative events

		% (n/total)
Inhospital		
MACE		4.9 (5/103)
Death		1.0 (1/103)
	Cardiac	1.0 (1/103)
	Non-cardiac	0.0 (0/103)
AMI		3.9 (4/103)
	With no ST segment elevation	3.9 (4/103)
	With ST segment elevation	0.0 (0/103)
MR		0.0 (0/103)
	Of the target vessel	0.0 (0/103)
	Of non-target vessel	0.0 (0/103)
Thrombosis of the stent		1.0 (1/103)
	CVA	0.0 (0/103)
	Vascular complications	0.0 (0/105)
30 days		
MACE		5.8 (6/103)
Death		1.9 (2/103)
	Cardiac	1.9 (2/103)
	Non-cardiac	0.0 (0/103)
AMI		3.9 (4/103)
MR		1.0 (1/103)
	Of target vessel	0.0 (0/103)
	Of target lesion	0.0 (0/103)
	Of non-target vessel	1.0 (1/103)
Thrombosis of the stent		1.0 (1/103)
CVA		0.0 (0/103)
12 months		
MACE		11.7 (12/103)
Death		5.8 (6/103)
	Cardiac	2.9 (3/105)
	Non-cardiac	2.9 (3/105)
AMI		4.9 (5/103)
MR		6.8 (7/103)
	Of the target vessel	3.9 (4/103)
	Of the target lesion	3.9 (4/103)
	Of the non-target lesion	2.9 (3/103)
Thrombosis of the stent		1.0 (1/103)
CVA		1.0 (1/103)

MACE: major adverse cardiac events; AMI: acute myocardial infarct; MR: myocardial revascularization; CVA: cerebrovascular accident.

At 12 months, MACE occurred in 12 patients, 6 allcause deaths (3 of cardiac death and 3 of non-cardiac death), 5 cases of non-STEMI, and 4 patients has TVR. However, it is important to point out the low incidence of stent thrombosis in the present study, occurring in only 1 patient; which was an acute stent thrombosis <24 hours after stenting. There were no cases of late or very late thrombosis.

**Figure 1:**
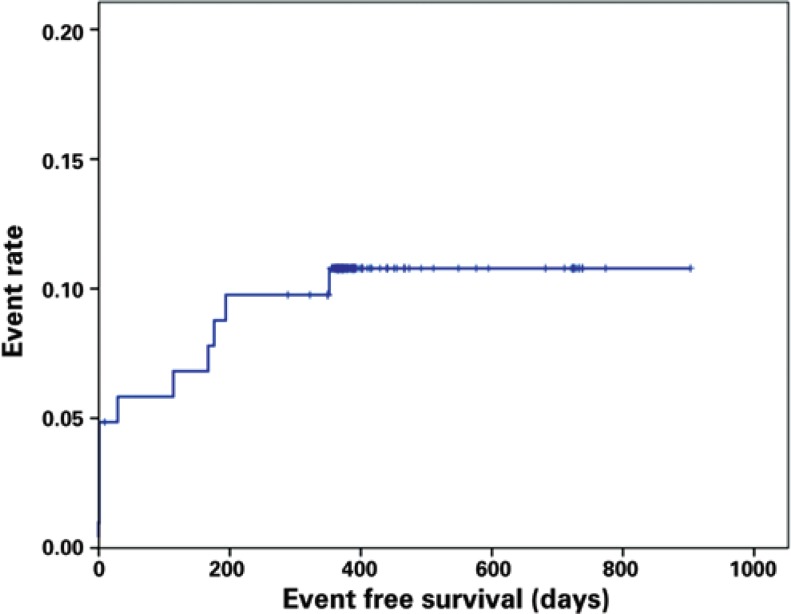
Kaplan-Meier curve demonstrating rate of events in all patients

**Figure 2:**
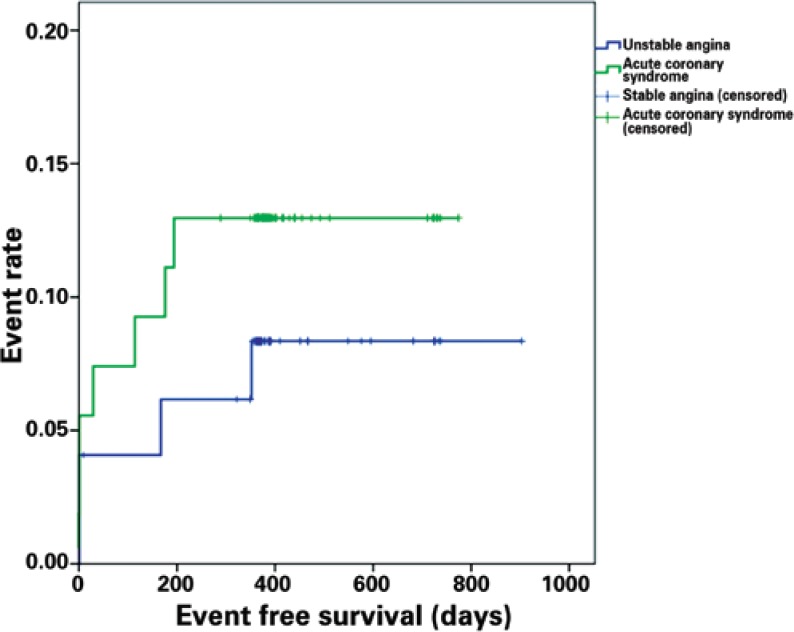
Kaplan-Meier curve demonstrating rate of events in the group of patients with stable angina and acute coronary syndrome

**Figure 3:**
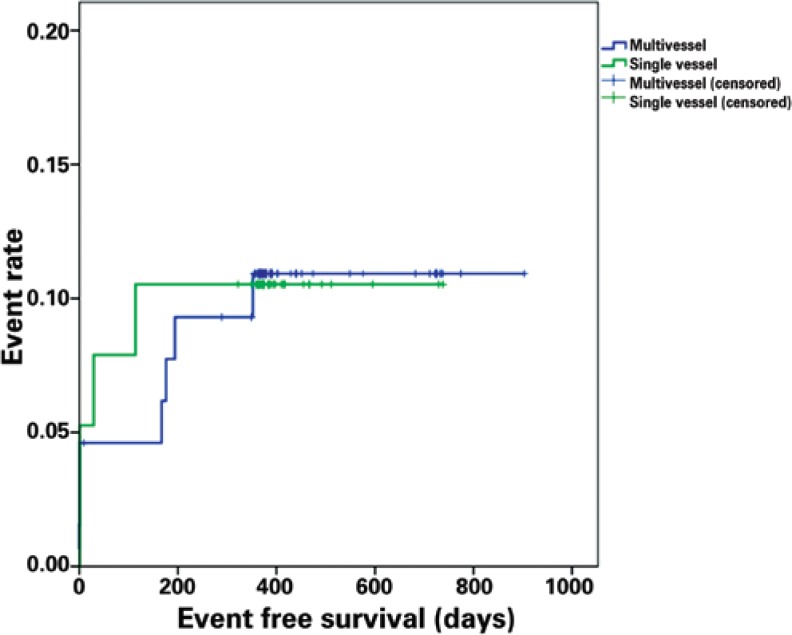
Kaplan-Meier curve demonstrating rate of events in the single arterial and multiple arterial group

Figure 1 shows Kaplan-Meyer curves in the overall population. Of note, albeit with no statistical significance, there was an trend toward between patients with acute coronary syndrome versus those with stable angina (87.0% *versus* 91.8%; p=0.43) (Figure 2). In addition, there was no difference in terms of event-free survival between the groups of patients with single versus multivessel disease (89.5% *versus* 89.2%; p=0.97) (Figure 3).

## DISCUSSION

The results of the present manuscript corroborate and add new data to those previously published on the use of the Biomatrix™ biolimus A9-eluting ^(9,11–17)^ stent. The main findings of this registry are: in a “real world”, all comers patients – including those with clinical (diabetes) and anatomical (multivessel, bifurcation, instent restenosis, and STEMI) complexity – the use of Biomatrix™ stent was safe and effective at long-term follow-up.

The presence of a durable polymer with a lack of biocompatibility in first-generation DES was associated with inflammation and vascular hypersensitivity reaction delay in reendothelization, and most importantly, with late and very late stent thrombosis and death. Biolimus is a synthetic drug analog of sirolimus, with a ten-fold higher lipophilicity than sirolimus, but with similar antiproliferative profile. The polylactic acid polymer is applied only on the abluminal surface, and after total release of the drug, it is completely dissolved into carbonic gas and water over a period of 6 to 9 months. These pharmacodynamics and bioengineering advantages make it, in thesis, a safer platform than the durable polymers used in first generation DES. The phase II study, STEALTH^(11)^ by Grube et al., was the first to evaluate the safety and efficacy of the Biomatrix™ stent. In a randomized and double blind study, enrolling 120 patients showed an extremely favorable results for the Biomatrix™ arm (intra-stent loss late luminal of 0.26mm) when compared with the control arm (0.74mm). The event-free survival at 6 months was similar between the two groups. These results have encouraged the development of a new clinical trial, LEADERS^(9)^, which randomized, 1,707 patients in ten centers in Europe. In this study, the safety and efficay of the Biomatrix™ (BES group) were compared to those of a first-generation sirolimus-eluting stent with a durable polymer (Cypher SELECT™, Cordis, Miami Lakes, FL, USA). At 9 months, the rates of MACE (cardiac death, AMI, or TVR) in the BES group was non-inferior to those treated sirolimus-eluting stent (SES). Additionally, BES was also not angiographicaly inferior to SES (instent stenosis of 20.9% *versus* 23.3%, respectivaly). This early clinical outcome reported similar rates of acute and subacute definitive stent thrombosis (1.6% for BES *versus* 1.6% for SES), as well as late and very late stent thrombosis (0.2% for BES *versus* 0.5% for SES). Our present analysis showed rates of ischemic events similar to the LEADERS study, although our data included more diabetic patients (1/3 *versus* 25% in the LEADERS), more STEMI, and small vessels (2.4mm *versus* 2.6 mm in the LEADERS). Remarkably, we observed only one case of early stent thrombosis (related to the procedure) and no cases of late or very late stent thrombosis. Thus, despite the small sample size in this registry, the present study suggests that this novel Biomatrix™ platform with a biodegradable polymer is safe even in higher-risk patients. Nevertheless, due to an incremental rates of stent thrombosis over time with the first-generation of DES, longer term follow-up is necessary to confirm the non-inferiority and/or superiority of the DES with biodegradable polymer Indeed, 4-year follow-up of the LEADERS^(12)^ study, showed once again non-inferiority of the biolimuseluting stent relative to the primary outcome for BES (18.7%) *versus* SES (22.6%). Additionally, there was a 38% relative risk reduction of definite stent thrombosis between one and four years of follow-up in the BES group compared with the SES group. Biomatrix™ safety superiority compared with first-generation DES may be explained by more strut stent coverage on long-term follow-up, as demonstrated by optical coherence tomography^(13)^.

Importantly, the Biomatrix™ stent also had its performance and safety evaluated in specific subgroups of patients. When patients were divided according to the Syntax Score (SX) into 3 groups (low SX if ≤8, intermediate SX if >8 and ≤16, and high SX if >16), the following rates of MACE were noted after two years follow-up: 9.4% for low SX, 12.0% for intermediate SX, and 18.4% for high SX (p<0.01). The rate of cardiac death in patients with high SX was significantly higher than the other two groups (7% *versus* 2.4% *versus* 1.8%). Nevertheless, in a subgroup of patients with greater anatomical complexity, those patients treated with BES had significantly lower rates of cardiac death when compared to the group treated with SES^(14)^. Conversely, in a subgroup of small vessels (reference diameter <2.75mm), there was no significant difference in the rates of MACE and in the rates of TVR between the 2 groups^(15)^. Finally, when analyzing STEMI patients, there was a significant reduction in MACE in patients treated with BES compared to those with SES (8.1% *versus* 19.3%)^(16)^_._

In a study by Soares Junior et al.^(17)^, assessing long-term follow-up in 100 patients (164 lesions) treated with Biomatrix™ stent (mean clinical follow-up of 243 days), the authors reported similar results to our analysis. In their study, the primary endpoint occurred in 9% of the patients (cardiac death in 4%, non-fatal AMI in 2%, and TVR in 3%), and the rate of stent thrombosis was 1%. Likewise, no case of late or very late thrombosis has been reported in this cohort of patients.

Recently, the COMPARE II^(18)^ study showed the safety and efficacy of biolimus-eluting stent with the biodegradable polymer (Nobori™, Terumo Corporation, Tokyo, Japan) compared with new-generation everolimuseluting stent with durable polymer (Xience V™, Abbot Vascular, Santa Clara, CA, USA). The positive safety endpoints of the biolimus-eluting stent of this large-scale and randomized trial open a new perspective for dual antiplatelet therapy <1 year, even including higher-risk patients.

The present study has the following limitations. It is an observational study, performed in a single center, without active control group. The present study sample size is small and the results should be considered exploratory only. Further studies are warranted to drawn definite analysis. The treatment of patients biolimuseluting stent was at physicians' discretion and, therefore, it is subject to selection. Finally, a longer clinical follow-up (>1 year) is required since the minority of patient had follow-up over one year. Therefore, the present data on safety and efficacy of biolimus-eluting stent cannot be extrapolated to those patients with longer follow-up period.

## CONCLUSIONS

Novel generation biolimus A9-eluting stent with biodegradable polymer is safe and effective in a real world, all-comers patients including those with high clinical and anatomical complexity, presenting low ischemic event rates on long-term-follow-up.
